# Expression and prognostic value of Cripto‐1 in early non‐small cell lung cancer

**DOI:** 10.1111/crj.13680

**Published:** 2023-08-01

**Authors:** Jian Zhong, Li Li, Qian Zhang, Jue Zou, Wei Liu, Chun Hua Xu

**Affiliations:** ^1^ Department of Thoracic Surgery Affiliated Nanjing Brain Hospital, Nanjing Medical University Nanjing China; ^2^ Department of Respiratory Medicine Affiliated Nanjing Brain Hospital, Nanjing Medical University Nanjing China; ^3^ Clinical Center of Nanjing Respiratory Diseases and Imaging Nanjing China; ^4^ Department of Pathology Affiliated Nanjing Brain Hospital, Nanjing Medical University Nanjing China

**Keywords:** Cripto‐1, NSCLC, prognosis, recurrence

## Abstract

**Objective:**

We aim to explore the expression of Cripto‐1 (CR‐1) protein in patients with early stage non‐small cell lung cancer (NSCLC).

**Methods:**

We investigated CR‐1 expression status in specimens obtained from 240 patients with resected NSCLC and 30 cases of para‐carcinous normal lung tissues.

**Results:**

Compared with normal lung tissue, the positive expression of CR‐1 protein in NSCLC was significantly increased (*p* < 0.005). Cox multivariate regression analysis showed that the expression of CR‐1 protein was an independent prognostic factor for early stage NSCLC (*p* = 0.002).

**Conclusion:**

Detecting CR‐1 protein can predict the prognosis and recurrence in patients with NSCLC.

## Graphical abstract

The positive expression of CR‐1 protein in early stage NSCLC was higher than that in normal lung tissue. Cox multivariate regression analysis showed that the expression of CR‐1 protein was an independent prognostic factor for early stage NSCLC. Detecting the expression of CR‐1 protein is helpful to predict the prognosis and recurrence of early stage NSCLC patients.

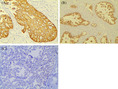

## 1 INTRODUCTION

Lung cancer is one of the highest morbidity and mortality.^1^ Lung cancer can be divided into NSCLC and SCLC, of which NSCLC accounts for 80%–85%.^2^ The 5‐year overall survival rate of early NSCLC was 71.7%, and 30%–40% of patients still had poor prognosis.^3^ For patients with high risk factors, such as poor differentiation, visceral pleural, vascular invasion and so on, adjuvant chemotherapy is recommended.^4^ Even with chemotherapy, 5‐year survival was only 60%–90% because of recurrence.^5^ It is a significant work to find high‐risk patients for chemotherapy, avoid unnecessary chemotherapy, and reduce the adverse prognosis caused by chemotherapy side effects.

Cripto‐1 (CR‐1) is a member of the cripto‐FRL1 family of epidermal growth factors.^6^, ^7^ Many studies have shown that CR‐1 is associated with the metastasis of many human tumours carcinomas.^8^, ^9^, ^10^, ^11^, ^12^, ^13^, ^14^, ^15^, ^16^, ^17^ Recent studies suggest that serum CR‐1 may be a biomarker for the diagnosis of lung cancer.^18^, ^19^ In addition, it was found that CR‐1 was overexpressed in lung tumours, and its high expression was related to lymph node metastasis and advanced disease stage, indicating its important role in lung cancer progression and metastasis.^20^ In lung cancer, few studies reported the association between CR‐1 expression and poor prognosis in early NSCLC patients.^21^ The significance of CR‐1 in early stage lung cancer remains to be investigated.

The purpose of this study was to explore the relationship between CR‐1 pretein and clinical features of NSCLC and to determine whether it is a prognostic marker for early stage NSCLC.

## 2 MATERIALS AND METHODS

### 2.1 Patients

A total of 240 patients with early stage NSCLC who underwent complete lung cancer resection and systemic lymph node dissection at thoracic surgery were enrolled in this study. In addition, 30 pairs of normal tissue were obtained 3 cm away from the cancerous tissue. All patients diagnosed with NSCLC according to the 2015 World Health Organization (WHO) classification of lung tumours were staged according to the eighth edition of the tumour lymph node metastasis (TNM) classification. None of the patients in the study received adjuvant therapy, including chemotherapy or radiation. Overall survival was defined as from the date of surgery to the date of death or the last follow‐up.

### 2.2 Immunohistochemistry

Immunohistochemical analysis was performed on tissue sections fixed, paraffin‐embedded and 4‐μm thick by antibiotic protein‐biotin‐peroxidase complex. Section was dewaxed and dehydrated with ethanol solution. Under the action of 0.3% catalase and methanol, the activity of endogenous peroxidase stopped for 20 minutes. After washing in phosphate buffer, slices were treated with 0.01‐M citrate buffer (pH 6.0). Irradiated slices in a microwave for 20 minutes and cooled at room temperature, incubated with a single antibody before 4°C and then received a second antibody. The results were shown by diaminobenzidine. In the immunohistochemical test, the negative control group was stained without primary antibody.

The expression of CR‐1 was evaluated by two experienced pathologists. The expression of CR‐1 was quantified by staining degree and intensity. Staining intensity score was 0 for no expression, 1 for weak expression, 2 for moderate expression and 3 for strong expression. The percentage of positive cells was scored as follows: 0 < 1%, 1 = 1%–33%, 2 = 33%–67%, 3 = 67%–100%. Multiply the percentage and intensity scores to get the final score. A score of 0–3 was considered negative expression, while a score above 3 was considered positive expression.^22^

### 2.3 Follow‐up

All patients were carefully followed up. The time of recurrence was recorded. The prognostic analysis included patients who died of recurrence after surgery. The median follow‐up was 58 months (range: 23 to 100 months).

### 2.4 Statistical analysis

SPSS 20 statistical software was applied to statistical analysis. The relationship between the expression of CR‐1 and clinicopathological characteristics was analysed by *χ* ^2^ test. Survival rate was calculated with Kaplan–Meier method and survival difference was compared by log‐rank test. Cox regression multivariate analysis was applied to determine independent prognostic factors. A p‐value of <0.05 was considered statistically significant.

## 3 RESULTS

### 3.1 Clinicopathological characteristics

The mean patient age was 66 years (42–78) for women and 65 years (39–76) for men. The NSCLC included 73.3% lung adenocarcinomas and 26.7% lung squamous cell carcinomas. 71.7% of patients had stage I lung cancer and 28.3% had stage II lung cancer. Sixty cases (25.0%) were well differentiated, 108 cases (45.0%) were moderately differentiated, and 72 cases (30.0%) were poorly differentiated. The clinicopathological features of the patients were summarized (Table 1 **)**.

**TABLE 1:** Clinicopathological characteristics of NSCLC patients.

Characteristic	*N*	%
Age (years)		
Range	39–78	
Mean	66.5	
Gender		
Male	96	40.0
Female	144	60.0
Smoking condition		
Non‐smoker	180	75.0
Smoker	60	25.0
Histological type		
SCC	64	26.7
ADC	176	73.3
Tumour differentiation		
Well	60	25.0
Moderate	108	45.0
Poor	72	30.0
TNM stage		
I	172	71.7
II	68	28.3

Abbreviations: ADC, adenocarcinoma; NSCLC, non‐small cell lung cancer; SCC, squamous cell carcinoma; TNM, tumour lymph node metastasis.

### 3.2 CR‐1 expression in lung cancer and normal lung tissues

**FIGURE 1:**
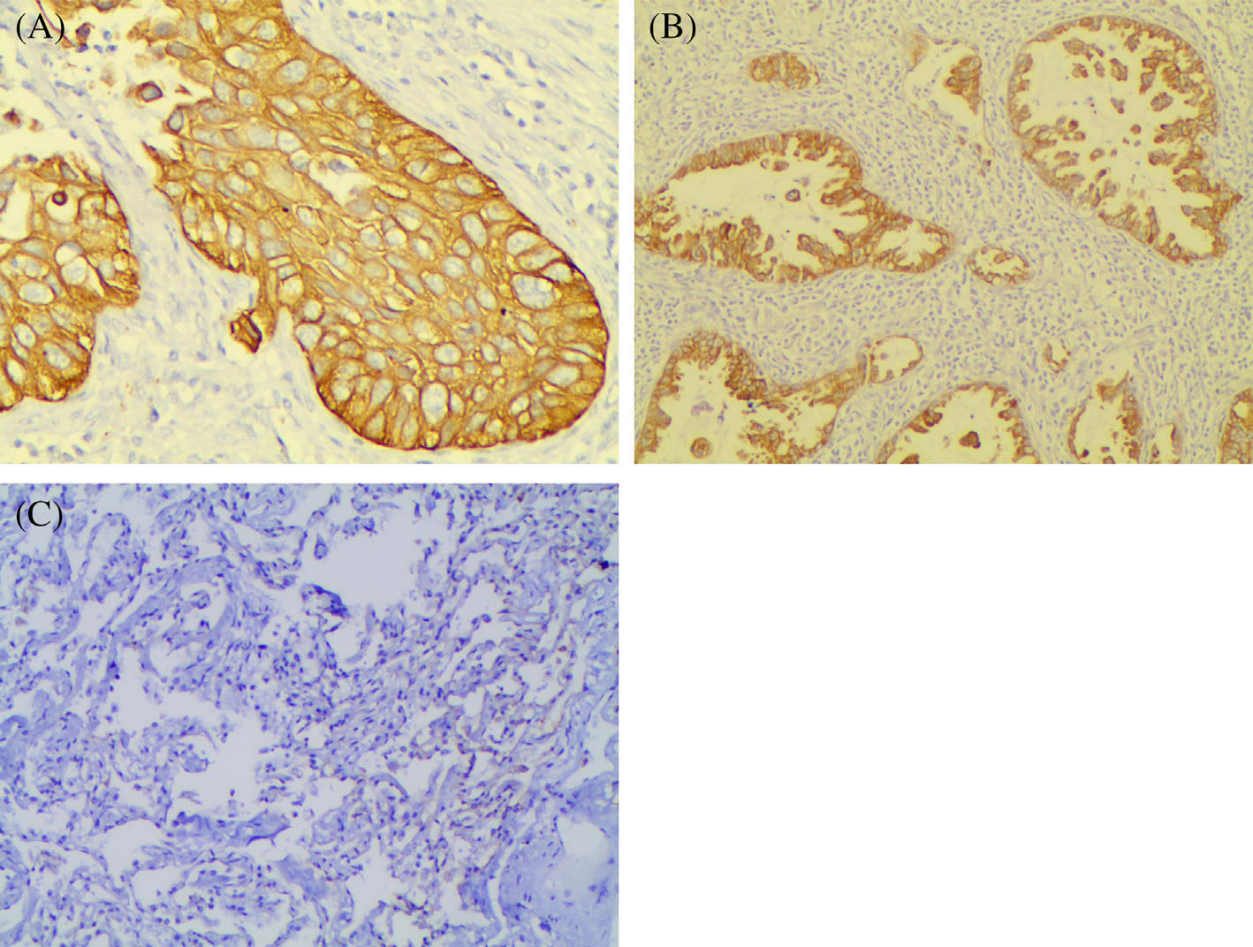
Immunohistochemical staining for CR‐1 protein expression. Positive staining of the cell cytoplasm in squamous cell carcinoma (A) and in adenocarcinoma (B). Negative staining of normal lung tissues (C). Original magnification ×400.

Specific CR‐1 staining was mainly located in the cytoplasm of tumour cells (Figure 1). Among 240 samples, 136 (56.7%) showed positive expression of CR‐1 protein. CR‐1 protein was expressed in 4 of 30 adjacent lung specimens (*p* < 0.001).

### 3.3 Association between CR‐1 expression and clinicopathological characteristics

The results showed the positive CR‐1 expression was associated with low differentiation and advanced stage of cancer (*p* = 0.028, 0.024, respectively). However, there was no correlation with sex, age, smoking status and pathology (Table 2).

**TABLE 2:** Association between CR‐1 expression and clinicopathologic characteristics.

Characteristics	Patients	No. of patients		
		CR‐1 positive	CR‐1 negative	*p*
Gender				0.349
Male	96	60	36	
Female	144	76	68	
Age (years)				0.196
<60	110	70	40	
≥60	130	66	64	
Smoking condition				0.287
Non‐smoker	180	96	84	
Smoker	60	40	20	
Histological type				0.145
SCC	64	44	20	
ADC	176	92	84	
Tumour differentiation				0.028*
Well‐moderate	168	84	84	
Poor	72	52	20	
TNM stage				0.024*
I	172	86	86	
II	68	50	18	

Abbreviations: ADC, adenocarcinoma; SCC, squamous cell carcinoma; TNM, tumour lymph node metastasis.

* Statistically significant difference (*p* < 0.05).

### 3.4 CR‐1 expression and tumours recurrence

All patients were followed up. The recurrence rate during follow‐up was 94 of the 240 patients (39.2%), 74 of whom had CR‐1 protein positive expression (54.4%) and 20 of whom had CR‐1 protein negative expression (19.2%) (*p* < 0.001, Table 3).

**TABLE 3:** Recurrence in 240 postoperative patients.

Group	Recurrence (*n*)	No recurrence (*n*)	Total (*n*)	*p*
Positive CR‐1 expression	74	62	136	<0.001
Negative CR‐1 expression	20	84	104	
Total	94	146	240	

### 3.5 Correlation of CR‐1 expression with overall survival

**FIGURE 2:**
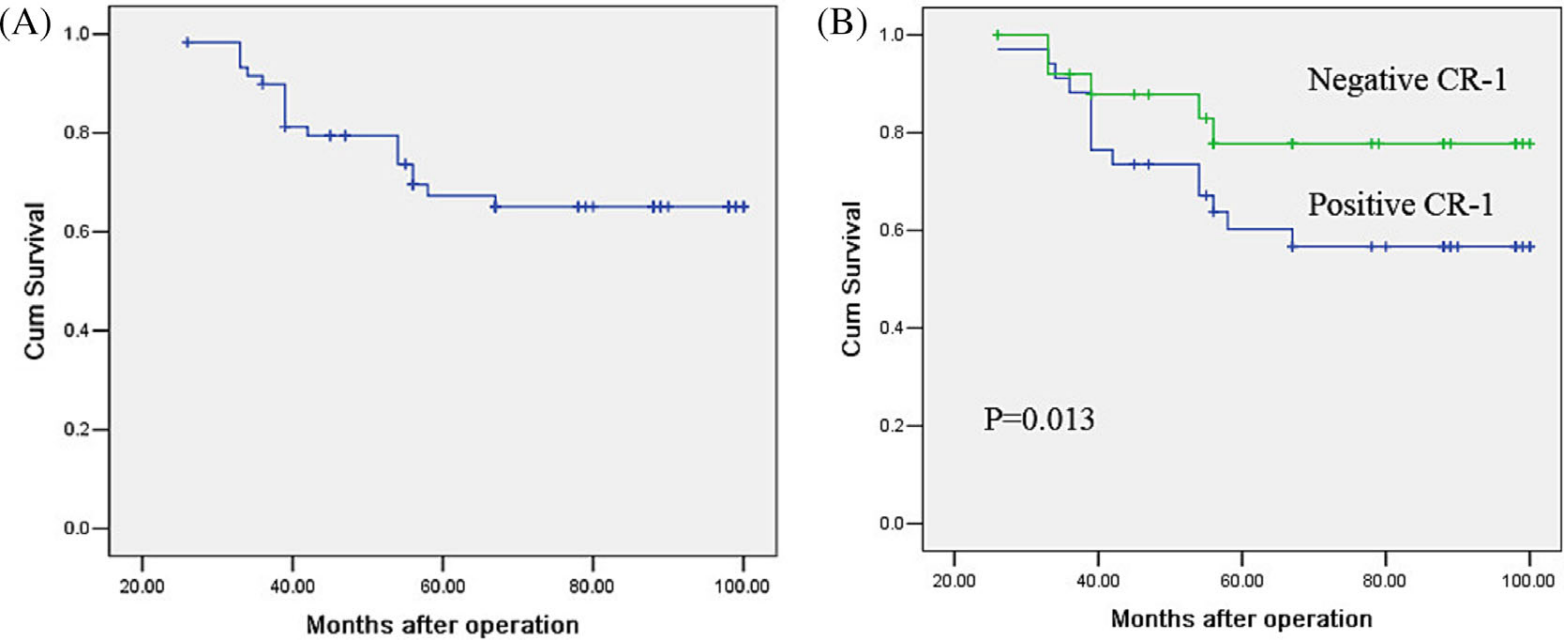
Overall survival of all patients (A) and patients with different CR‐1 status (B).

Patients who survived the last follow‐up had an average of 58 months of overall survival. The overall five‐year survival rate was 69.2%, 58.8% in patients with positive CR‐1 expression and 82.7% in patients with negative CR‐1 expression (Figure 2A,B).

In multivariate analysis for overall survival, the significant predictor was more CR‐1 expression (*p* = 0.002). Advanced stage and poor differentiation were associated with overall survival. We also analysed prognostic factors using multivariate analysis for the survival. The results analysis revealed that differentiation and CR‐1 were independent prognostic factors (Table 4).

**TABLE 4:** Multivariate analysis of prognostic variables for overall survival.

Variable	Overall survival	
	HR (95% CI)	*p*
Gender (male vs. female)	1.432 (0.670–3.062)	0.354
Age (<60 vs. ≥60)	1.731 (0.633–4.737)	0.285
TNM stage (I vs. II)	5.226 (1.575–17.338)	0.007
Differentiation (well‐moderate vs. poor)	2.964 (1.642–7.926)	0.016
Histological type (SCC vs. ADC)	1.011 (0.965–1.109)	0.231
CR‐1 expression (Positive vs. Negative)	5.331 (1.833–15.507)	0.002

Abbreviations: ADC, adenocarcinoma; CI, confidence interval; HR, hazard ratio; SCC, squamous cell carcinoma; TNM, tumour lymph node metastasis.

## 4 DISCUSSION

This study found that 56.7% of early NSCLC expressed CR‐1. The key to determining whether patients should undergo adjuvant chemotherapy after surgery is to improve the survival rate of high‐risk patients and reduce unnecessary chemotherapy toxicity. Our study suggested that CR‐1 could play a role in postoperative NSCLC recurrence. CR‐1 may be biomarker for evaluating adjuvant chemotherapy.

Studies have shown that CR1 protein was implicated in the invasion and metastasis potential of many human cancers. The overexpression of CR‐1 protein in tumours may promote cell proliferation, invasion, migration and tumour angiogenesis.^23^ As far as we know, there is no relevant study on the correlation between CR‐1 expression and early stage NSCLC. Xu et al detected the CR‐1 mRNA in NSCLC and reported that the CR‐1 expression was associated with lung cancer progression.^20^ Our results indicated that the CR‐1 expression was high in early stage NSCLC tissues. In addition, CR‐1 overexpression was associated with differentiation and stage. There was no correlation between overexpression of CR‐1 protein and gender, age, smoking status and pathological type.

Previous studies have shown that elevated serum CR‐1 levels in NSCLC patients are associated with poor prognosis.^18^ Our study showed that the 5‐year survival rate of patients with positive CR‐1 was significantly lower than that of patients with negative CR‐1. The 5‐year overall survival rate was 69.2% in 120 NSCLC patients, 58.8% in CR‐1 positive expression patients and 82.7% in CR‐1 negative expression patients. Multivariate analysis confirmed that the expression of CR‐1 protein was an independent prognostic factor.

The mechanism of CR‐1 in the process of metastasis remains unclear. It has been shown that CR‐1 can mediate signal transduction of other TGF‐beta ligands. The binding of CR‐1 to TGF‐β1 and Activin can inhibit TGFβ‐1 and Activin signalling in mammalian cells.^24^ Moreover, CR‐1 can activate the PI3‐K/AKT/GSK‐3β and ras/raf/MAPK signalling pathways independently of Nodal and ALK4.^25^ The study showed that the Wnt/B‐catenin/LEF‐1 signalling pathway may intersect with CR‐1 signalling pathway to regulate cell adhesion and migration.^26^ Strizzi et al reported that CR‐1 may promote the expression of signalling molecules associated with epithelial‐mesenchymal transformation.^27^

In conclusion, our results suggest that CR‐1 overexpression is significantly associated with cancer recurrence. These results suggest that CR‐1 may be a predictor and prognostic factor of NSCLC recurrence in early stage NSCLC.

## AUTHOR CONTRIBUTIONS

ChunHua Xu, Jian Zhong and Jue Zou designed the study. Li Li and Qian Zhang collected data. Chunhua Xu and Wei Liu wrote the paper.

## CONFLICT OF INTEREST STATEMENT

The authors declare no conflict of interest.

## ETHICS STATEMENT

The study was approved by the Ethics Committee of the Affiliated Nanjing Brain Hospital, Nanjing Medical University. All patients expressed informed consent.

## Data Availability

The data that support the findings of this study are available from the corresponding author upon reasonable request.
